# Tissue-Specific Mitochondrial Functionality and Mitochondrial-Related Gene Profiles in Response to Maternal Nutrition and One-Carbon Metabolite Supplementation During Early Pregnancy in Heifers

**DOI:** 10.3390/ani15182689

**Published:** 2025-09-14

**Authors:** Kazi Sarjana Safain, Matthew S. Crouse, Mara R. Hirchert, Yssi L. Entzie, Jessica G. Syring, Mojtaba Daneshi, Muhammad Anas, Layla E. King, Lawrence P. Reynolds, Pawel P. Borowicz, Carl R. Dahlen, Alison K. Ward, Joel S. Caton, Kendall C. Swanson

**Affiliations:** 1 Department of Animal Sciences, Center for Nutrition and Pregnancy, North Dakota State University, Fargo, ND 58105, USA; kazi.safain@ndsu.edu (K.S.S.); mara.r.hirchert@ndsu.edu (M.R.H.); yssi.entzie@ndsu.edu (Y.L.E.); jessica.syring@ndsu.edu (J.G.S.); mojtaba.daneshi@ndsu.edu (M.D.); muhammadanas2365@gmail.com (M.A.); larry.reynolds@ndsu.edu (L.P.R.); pawel.borowicz@ndsu.edu (P.P.B.); carl.dahlen@ndsu.edu (C.R.D.); kendall.swanson@ndsu.edu (K.C.S.); 2 United States Department of Agriculture, Agriculture Research Service, U.S. Meat Animal Research Center, Clay Center, NE 68933, USA; matthew.s.crouse@gmail.com; 3 Department of Agriculture and Natural Resources, University of Minnesota Crookston, Crookston, MN 56716, USA; king0635@crk.umn.edu; 4 Department of Veterinary Biomedical Sciences, University of Saskatchewan, Saskatoon, SK S7N 5A2, Canada; alison.ward@usask.ca

**Keywords:** one-carbon metabolism, mitochondria, maternal nutrition, metabolism, transcriptome, fetal development, beef heifers

## Abstract

Background: Mitochondria are essential for fetal development, regulating energy metabolism and metabolic programming. This study examined how maternal nutrition and one-carbon metabolite (OCM) supplementation during early gestation affect mitochondrial function in fetal liver and muscle at day 161 of gestation in beef heifers. Methods: Twenty-nine crossbred Angus heifers were assigned to one of four treatments in a 2 × 2 factorial design: control (CON; 0.45 kg/day ADG) or restricted gain (RES; −0.23 kg/day), with or without OCM supplementation. Treatments were applied from breeding to day 63 of gestation, after which all heifers received a common diet. Fetal liver and muscle tissues were collected at day 161. Mitochondrial respiration (Seahorse assay), mtDNA copy number (qPCR), and mitochondria-related gene expression (RNA-seq) were assessed. Results: In fetal liver, state 3 respiration was highest in CON + OCM, while state 4o respiration was lowest in RES + OCM (*p* ≤ 0.05). mtDNA copy number was greater in RES and +OCM groups. In fetal muscle, mtDNA copy number was influenced by gain, but respiration was unaffected. Transcriptomic analysis revealed more mitochondria-related differentially expressed genes (mtDEGs) in fetal muscle than liver (90% versus 10% of total mtDEG), with most genes downregulated in the RES and +OCM groups compared to the CON and −OCM groups (FDR ≤ 0.10). Conclusions: OCM supplementation enhanced mitochondrial respiration and biogenesis in fetal liver, likely via post-translational mechanisms. In contrast, fetal muscle showed downregulation of mitochondria-related genes without functional changes, indicating transcriptional reprogramming with potential effects on later metabolic function. These results underscore early gestation as a critical window for OCM-based nutritional interventions to improve metabolic outcomes in livestock.

## Simple Summary

Mitochondria are essential for fetal development because they regulate energy production and play a key role in long-term health. This study examined how maternal nutrition and supplementation with one-carbon metabolites (OCM)—including methionine, choline, folate, and vitamin B_12_—during early pregnancy influence fetal mitochondrial function in beef heifers at mid-gestation. Heifers were fed different diets during the first 63 days of pregnancy, varying in both their rate of weight-gain and whether they received OCMs. At day 161 of gestation, mitochondrial function and regulation were evaluated in fetal liver and muscle tissues. The fetal liver responded strongly to OCM supplementation and restricted maternal weight-gain, showing higher mitochondrial activity and increased mitochondrial DNA copy numbers—both signs of enhanced energy metabolism. These changes occurred without major shifts in gene expression, suggesting regulation may occur at the protein level. In contrast, fetal muscle showed no changes in mitochondrial activity but had reduced expression of genes involved in fat metabolism and energy production. This suggests early transcriptional changes that could affect muscle energy use after birth. These findings highlight early-pregnancy as a critical period when maternal nutrition can shape fetal organ development and function, with potential long-term effects on calf growth, metabolism, and productivity.

## 1. Introduction

Pregnancy represents a period of profound anatomical, physiological, and biochemical adaptations, beginning shortly after fertilization and continuing throughout gestation. These changes are driven by physiological stimuli from the developing fetus and placenta, placing significant metabolic demands on the maternal system [1]. In the fetus, mitochondria play a critical role in meeting the high energy demands of development by orchestrating cellular energy production through oxidative phosphorylation (OXPHOS) and supporting intermediary metabolism. Beyond energy production, fetal mitochondria are integral to processes such as lipid and amino acid metabolism, calcium homeostasis, and redox signaling, all of which are vital for cellular homeostasis and growth [2,3].

In the fetus, mitochondria are particularly significant during pregnancy, as they regulate growth and development by generating ATP, facilitating nutrient metabolism, and contributing to epigenetic modifications through the production of metabolites—such as S-adenosylmethionine (SAM), acetyl-CoA, and NAD+—that serve as essential cofactors for methylation and acetylation reactions. Their role becomes evident early in embryogenesis, as mitochondrial biogenesis and function increase to meet the metabolic demands of the developing conceptus [2]. In fetal liver and muscle, mitochondrial function supports nutrient metabolism and energy production, processes that are essential for organogenesis and tissue-dependent growth [4]. However, fetal mitochondria are highly sensitive to environmental factors, including maternal diet, which can disrupt mitochondrial DNA (mtDNA) synthesis, repair, and function, potentially leading to developmental abnormalities [5,6].

Maternal nutrition during gestation is a critical determinant of pregnancy outcomes, influencing fetal development through mechanisms that extend beyond nutrient supply [7]. Nutrients such as B vitamins, and amino acids are essential for mitochondrial DNA synthesis, methylation, and the proper functioning of the electron transport chain [8]. Deficiencies in these nutrients may impair mitochondrial function, contributing to oxidative stress and developmental defects, as seen in both humans [9] and livestock [10]. One-carbon metabolism, encompassing the folate and methionine cycles, is particularly crucial as it supplies methyl donors for DNA and histone methylation, which regulate gene expression and developmental programming [11]. Supplementing OCM such as methionine, choline, folate, and vitamin B_12_ has been shown to enhance mitochondrial function, improve metabolic capacity, and mitigate the effects of maternal nutrient restriction on fetal development [12]. Beyond their direct roles in mitochondrial function, nutrients derived from the maternal diet also serve as key metabolic intermediates that influence cellular processes at multiple levels. In ruminants, carbohydrates are fermented by rumen microbes into volatile fatty acids [13] (e.g., acetate, propionate, and butyrate), which are then absorbed and utilized in mitochondrial metabolism to generate essential metabolites such as ATP, acetyl-CoA, and NADH. These metabolites, in turn, have the potential to modulate the metabolome-(epi)genome axis, reactive oxygen species (ROS) production, lipid metabolism (e.g., de novo lipogenesis and β-oxidation), and insulin sensitivity, which can impact fetal development and long-term metabolic health [14]. Moreover, diet-derived mitochondrial metabolites, together with S-adenosylmethionine (SAM)—the primary methyl donor—have a prominent role in epigenetic modifications, further linking maternal nutrition to fetal programming and developmental outcomes, a phenomenon also evident in animals [15].

Ensuring the continuous cycling of the methionine and folate cycles requires the coordinated availability of multiple nutrients, as supplementing a single nutrient individually may not be sufficient to sustain the complex interdependencies of one-carbon metabolism [16,17]. The metabolism of choline, folate, vitamin B_12_, and methionine is intricately interrelated, and disturbances in one pathway often trigger compensatory adjustments in others [18]. Thus, comprehensive OCM supplementation during early gestation in cattle could mitigate the detrimental effects of maternal nutrient restriction by ensuring the availability of essential cofactors for chromatin remodeling, gene expression regulation, and mitochondrial function [11,19]. Despite this potential, there is a lack of studies investigating the interconnectedness of mitochondria, one-carbon metabolism, and fetal development in cattle, particularly in understanding how mitochondrial function adapts in response to maternal dietary interventions across gestation.

Emerging research suggests that mitochondrial adaptations during pregnancy are tissue-specific and influenced by stage of gestation [20,21]. The liver, as a primary site for nutrient processing and metabolic regulation, undergoes significant functional maturation during gestation [22], while muscle tissue prioritizes structural growth and myofiber development [23]. These differences underscore the importance of studying mitochondrial function and gene expression in a tissue-targeted context to better understand how maternal dietary interventions shape fetal development. Our previous research demonstrated that maternal OCM supplementation influenced metabolic profiles in fetal liver during early gestation (day 63) [24] but did not affect mitochondrial respiration [25]. However, the mid-to-late gestation period represents a phase of increased metabolic demand and mitochondrial biogenesis [26], which may amplify the effects of maternal nutrition on fetal tissues. Building on these findings, this study evaluates the lasting effects of maternal nutrition and OCM supplementation administered during early gestation (up to day 63) on fetal mitochondrial function at a later gestational stage (day 161). We hypothesized that restricted maternal nutrition would impair mitochondrial functionality and metabolic programming, and that OCM supplementation would mitigate these effects in a tissue-dependent manner, reflecting the distinct developmental roles of the fetal liver and muscle. By integrating mitochondrial respiration analysis, mtDNA quantification, and transcriptomic profiling, this study aimed to provide novel insights into the role of maternal dietary inputs in shaping fetal metabolic adaptations and long-term developmental programming.

## 2. Methods

### 2.1. Animals and Ethics

All procedures were approved by the North Dakota State University Animal Care and Use Committee. The study’s experimental design, treatments, and diets were previously detailed by Daneshi et al. [27]. In brief, a total of 72 Angus heifers were estrous synchronized using a 7-day Select Synch + CIDR protocol and artificially inseminated with female-sexed semen from a single sire to avoid sex-specific variation. Heifers on the control treatment (CON) were targeted for an average daily gain (ADG) of 0.45 kg/day (actual ADG: 0.60 kg/day) to reach 80% of mature BW by calving. Those on the restricted treatment (RES) were targeted to lose 0.23 kg/day (actual ADG: −0.23 kg/day). Their diet comprised corn silage, alfalfa hay, corn grain, alfalfa/grass hay, and vitamin/mineral premix (Trouw dairy VTM w/Optimins, Trouw Nutrition USA, Highland, IL, USA). Further details of the diet composition are presented in Table 1 and have been elaborated upon by Syring et al. [28]. Weekly weigh-ins and diet adjustments ensured targeted gains. Heifers were limit-fed to meet targeted ADG and individually fed daily at 0800 h using an electronic head gate system (American Calan; Northwood, NH, USA). At 35 days post-insemination, pregnancy was confirmed by transrectal ultrasonography detecting fetal heartbeats, and at day 63 fetal sex was determined by ultrasonography.

Heifers on the OCM-supplemented treatments (+OCM) received 7.4 g/day of rumen-protected methionine (Smartamine, Adisseo, China) and 44.4 g/d rumen-protected choline (ReaShure, Bal-chem Inc., New Hampton, NY, USA) in a corn carrier, plus weekly intramuscular injections of 20 mg of vitamin B_12_ (MWI Animal Health, Boise, ID, USA) and 320 mg of folic acid (Spectrum Chemical Mfg. Corp., New Brunswick, NJ, USA). Non-supplemented heifers (−OCM) received a corn carrier and weekly saline injections. Supplements were top-dressed using a fine ground corn carrier as detailed in Table 1. Treatments were administered until day 63 of gestation, after which all heifers were transitioned to the CON − OCM treatment, targeting a gain of 0.45 kg/day for the rest of the study. Ultrasound on day 63 identified fetal sex, and only female fetuses (*n* = 29, CON − OCM = 8, CON + OCM = 7, RES − OCM = 7, RES + OCM = 7) were included in subsequent stages. On day 161, heifers were harvested at the NDSU Meat Laboratory, and fetal liver and hindlimb muscle (semitendinosus) tissues were isolated.

### 2.2. Mitochondria Isolation

Mitochondria were isolated from fresh fetal liver and muscle tissues following a protocol adapted from Claycombe et al. [29]. Approximately 0.5 g of tissue was collected from both liver and muscle, minced, and homogenized in 10 mL of cold MSHE + 0.2% BSA + 4 mM ADP buffer (225 mM mannitol, 75 mM sucrose, 20 mM HEPES, 1 mM EGTA, 4 mM ADP and 0.2% BSA, pH 7.4) using a tissue homogenizer (Polytron PT 10/35) at low-medium speed on ice. The homogenate was centrifuged at 500× *g* for 10 min at 4 °C to remove debris. The supernatant was filtered through a 100 µm nylon mesh and centrifuged at 9900× *g* for 10 min at 4 °C. The resulting mitochondrial pellet was washed, resuspended in 10 mL of fresh MSHE + 0.2% BSA + 4 mM ADP buffer, and centrifuged again at 9900× *g* for 10 min. The final mitochondrial pellet was resuspended in 0.5 mL of MSHE + 0.2% BSA + 4 mM ADP buffer. The total protein concentration was then measured using the Pierce Detergent Compatible Bradford Assay (ThermoFisher, Rockford, IL, USA), following the manufacturer’s instructions.

### 2.3. Oxygen Consumption Rate (OCR) Determination

Mitochondrial oxygen consumption was evaluated using the Seahorse XFe24 Analyzer (Agilent Technologies, Santa Clara, CA, USA) with modifications to protocols from Rogers et al. [30]. The main modification was the addition of ADP (4 mM) directly into the 1X MAS media (220 mM mannitol, 70 mM sucrose, 10 mM KH_2_PO_4_, 5 mM MgCl_2_, 2 mM HEPES, 1 mM EGTA), used to dilute the isolated mitochondria. This allowed the assay to begin in State 3 respiration, which represents active ADP-stimulated respiration reflecting oxidative phosphorylation through complex II, as succinate was used as a substrate and rotenone (2 µM) was included to block complex I. Freshly isolated mitochondria were diluted in cold 1 × MAS + 0.2% (*w*/*v*) BSA + 4 mM ADP + 10 mM Na succinate + 2 µM rotenone (pH 7.2). Optimal mitochondrial amounts were determined through preliminary titration experiments testing different mitochondrial protein concentrations for each tissue type, resulting in the use of 10 µg for muscle and 5 µg for liver per well in XF24 plates (final volume of 50 µL), followed by centrifugation at 2000× *g* for 20 min at 4 °C for adherence.

The sensor cartridge was hydrated and calibrated per manufacturer guidelines. After centrifugation, 450 µL of pre-warmed (37 °C) assay buffer (1 × MAS + 0.2% BSA + 4 mM ADP + 10 mM Na succinate + 2 µM rotenone) was added to each well. The assay measured the following respiratory states: State 3 respiration: initiated by the presence of ADP, reflects active oxidative phosphorylation and ATP production; State 4o respiration: measured after injection of oligomycin (2.5 µg/mL final, Port A), represents proton leak or non-ATP-linked respiration (leak respiration), as oligomycin inhibits ATP synthase; State 3u respiration: induced by FCCP (4 µM final, Port B), an uncoupler that collapses the proton gradient, reflects maximal respiratory capacity of the electron transport chain; and Non-mitochondrial respiration: determined after antimycin A (4 µM final, Port C) injection, which inhibits complex III and halts mitochondrial electron flow, allowing correction for background oxygen consumption. Injection volumes were as follows: Port A, 55 µL oligomycin; Port B, 60 µL FCCP; and Port C, 65 µL antimycin A. The assay was conducted with five biological replicates and three measurement cycles per state. At least three replicates per treatment group were used for final analysis.

Analysis of OCR was performed using Seahorse Wave software v2.6 (Agilent Technologies, Santa Clara, CA, USA), and values were expressed as pmol O_2_/min/µg mitochondrial protein. Respiratory control ratios (RCR) were calculated as the ratio of State 3 to State 4o (State 3/State 4o) or State 3u to State 4o (State 3u/State 4o), reflecting mitochondrial coupling efficiency.

### 2.4. Relative Mitochondrial DNA Copy Number Determination

Total DNA was extracted from 25 mg of frozen fetal liver and muscle tissues using the DNeasy Blood & Tissue Kit (Qiagen, Hilden, Germany), following the manufacturer’s instructions. The DNA concentration was measured with the Qubit dsDNA BR Assay (Thermo Fisher Scientific, Waltham, MA, USA) and standardized to 40 ng/μL. Primers were designed for two mitochondrial genes: the NADH dehydrogenase subunit 2 (*ND2*) and cytochrome oxidase subunit III (*COX3*). The actin B (*ACTB*) gene was used as a nuclear control gene (Supplementary Table S1). Quantitative real-time PCR (qPCR) was performed using a QuantStudio™ 5 system (Thermo Fisher Scientific, Waltham, MA, USA). Each reaction was set up with 40 ng of genomic DNA, specific primers (500 nmol/L), and iTaq Universal SYBR Green Supermix (Biorad, Hercules, CA, USA) in a 10 µL final volume, run in triplicate. The PCR conditions were 95 °C for 5 min, followed by 35 cycles of 95 °C for 3 s and 59 °C for 30 s, with a melting curve analysis (65–95 °C) in the last cycle to evaluate amplification specificity. A standard curve was created using 10-fold serial dilutions (10^−1^ to 10^−8^) for each run. The relative mtDNA copy number was calculated with the following formula [31]: mtDNA copy number = 2^(1+(Ct_n_gene-Ct_mt_gene))^, where Ct represents the cycle threshold. Data from the two tissue types were analyzed separately.

### 2.5. RNA Extraction and Sequencing

Total RNA was extracted from fetal liver and muscle tissues collected in RNAlater^®^ solution (Life technologies, Carlsbad, CA, USA) using the RNeasy Plus Universal Mini Kit (Qiagen, Hilden, Germany) according to the manufacturer’s protocol. RNA concentration and quality were assessed with the Qubit RNA BR Assay (Thermo Fisher Scientific, Waltham, MA, USA). The RNA samples were then sequenced at NovoGene (Novogene, Sacramento, CA, USA). Differential expression analysis was performed using the QIAGEN RNA-seq Analysis Portal 5.0 (Qiagen, Aarhus, Denmark). From the complete list of differentially expressed genes, mitochondria-related genes (mtDEGs) were identified by referencing a previously published list of mitochondria-associated genes [32] and were selected for subsequent Gene Ontology (GO) and KEGG pathway analyses using ShinyGO 0.80 [33].

### 2.6. Statistical Analysis

Mitochondrial DNA copy number and mitochondrial respiration variables were analyzed with PROC MIXED (SAS 9.4). The experimental unit was the individual heifer. Fixed effects were nutritional plane (CON vs. RES), OCM supplementation (−OCM vs. +OCM), and their interaction (Gain × OCM). A *p*-value of 0.05 or less was considered statistically significant. For transcriptomic analysis, fold change (FC) and *p*-values were used to assess statistical significance, with false discovery rate (FDR) correction applied. mtDEGs were identified using an FDR-adjusted *p*-value threshold of <0.10, while GO and KEGG pathway analyses were conducted using an FDR-adjusted *p*-value threshold of <0.05. Fold changes are reported as log2(Group2/Group1). A positive value indicates higher expression in the second group relative to the first.

## 3. Results

### 3.1. Mitochondrial Respiration Profiles in Fetal Liver and Muscle Tissues

In fetal liver mitochondria (Table 2), State 3 respiration rates were influenced by the interaction between Gain and OCM supplementation (*p* = 0.004) such that the CON + OCM group exhibited greater state 3 respiration rate with RES − OCM being intermediate and equal to all treatment combinations. Similarly, state 4o respiration rates were affected by the Gain × OCM interaction (*p* = 0.04) where the CON + OCM group demonstrated a greater (*p* = 0.01) O_2_ consumption rate (78.1 pmol/min/µg protein) compared to the RES + OCM group (57.2 pmol/min/µg protein). No significant differences were observed in respiratory control ratio (RCR) across treatment groups (*p* > 0.05); however, OCM supplementation tended to increase (*p* = 0.08) RCR values in the +OCM compared to −OCM. On the contrary, in fetal muscle mitochondria (Table 3), neither State 3 respiration, state 4o respiration, nor RCR were different among treatment groups (*p* > 0.05).

### 3.2. mtDNA Copy Number Variations in Fetal Liver and Muscle

**Figure 1:**
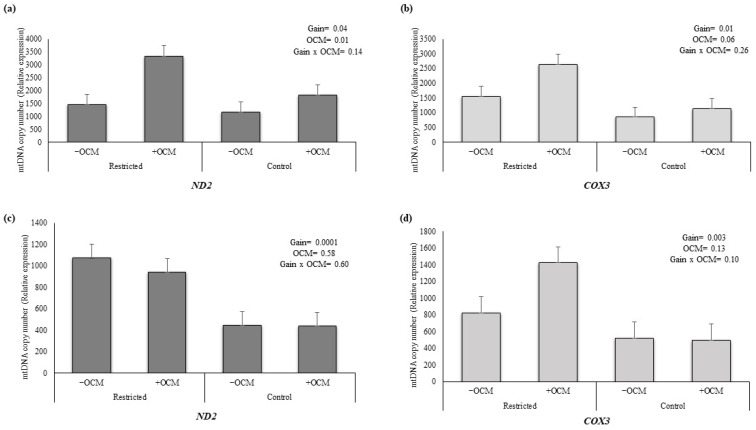
The mtDNA copy number variation in fetal liver and muscle tissues for *ND2* (NADH dehydrogenase subunit 2) and *COX3* (cytochrome oxidase subunit III) using qPCR. (**a**) mtDNA copy number for *ND2* in fetal liver, (**b**) mtDNA copy number for *COX3* in fetal liver, (**c**) mtDNA copy number for *ND2* in fetal muscle, and (**d**) mtDNA copy number for *COX3* in fetal muscle. Maternal treatments were administered from breeding (day 0) to day 63 of gestation in first-calf heifers: control gain (CON; 0.45 kg/d) vs. restricted gain (RES; −0.23 kg/d) and one-carbon metabolite supplementation (+OCM; methionine, choline, folate, vitamin B_12_) vs. no supplementation (−OCM). After day 63, all heifers received a common diet. Fetal liver and muscle were collected at day 161 of gestation.

The mtDNA copy number for *ND2* in fetal liver (Figure 1a) was influenced by both Gain (*p* = 0.04) and OCM supplementation (*p* = 0.01), with no significant interaction between the two factors (*p* = 0.14). Similar results were observed for *COX3* (Figure 1b), with a significant effect of Gain (*p* = 0.01) and a trend for OCM supplementation (*p* = 0.06). For both genes, mtDNA copy numbers were greater in the RES compared to CON, and OCM supplementation resulted in greater mtDNA copy number than no supplementation.

In fetal muscle, mtDNA copy numbers for both *ND2* and *COX3* (Figure 1c,d) followed a similar pattern, with higher levels observed in the RES group compared to the CON group (*p* = 0.0001 for *ND2*; *p* = 0.003 for *COX3*). Neither OCM supplementation nor the interaction between Gain and OCM affected mtDNA copy numbers for both genes (*p* > 0.05).

### 3.3. Mitochondria-Related Differentially Expressed Genes (mtDEGs) in Fetal Liver

Transcriptomic analysis revealed only two mitochondria-related differentially expressed genes (mtDEGs) in fetal liver tissues (Table 4). *RPL12* was consistently identified in three pairwise comparisons and was upregulated, with a ~1500-fold increase in the CON + OCM, RES + OCM, and RES − OCM groups compared to the CON − OCM group (FDR ≤ 0.10). Additionally, *CYP3A5_2* was observed as a liver mtDEG (FDR ≤ 0.10), with an 8-fold lower expression in the RES + OCM group compared to the CON − OCM and RES − OCM groups. Due to the limited number of mtDEGs identified in the fetal liver, no further analysis was performed. Complete mitochondria-related gene expression tables for all pairwise comparisons for fetal liver are provided in Supplementary File S1.

### 3.4. Mitochondria-Related Differentially Expressed Genes (mtDEGs) in Fetal Muscle

**Figure 2:**
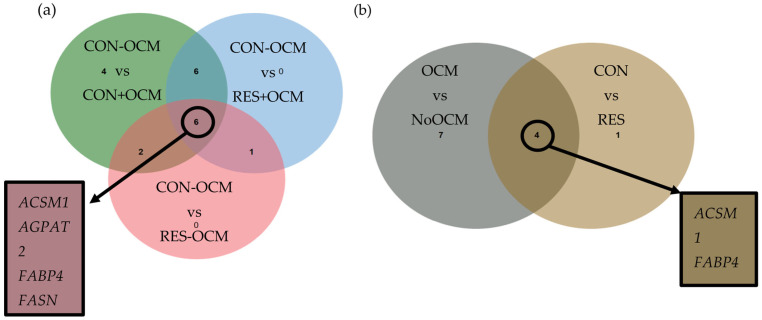
Mitochondria-related differentially expressed genes (mtDEGs) in fetal muscle at day 161 of gestation. Maternal treatments were administered from breeding (day 0) to day 63 of gestation in first-calf heifers: control gain (CON; 0.45 kg/d) vs. restricted gain (RES; −0.23 kg/d) and one-carbon metabolite supplementation (+OCM; methionine, choline, folate, vitamin B_12_) vs. no supplementation (−OCM) (**a**) Pairwise contrasts among treatment combinations. Each circle denotes a contrast (e.g., CON − OCM vs. CON + OCM, CON − OCM vs. RES + OCM, CON − OCM vs. RES − OCM). Numbers indicate mtDEG counts at FDR (q-value) ≤ 0.10; overlaps show genes shared across contrasts. Callouts list representative shared downregulated genes (e.g., *ACSM1*, *AGPAT2*, *FABP4*, *FASN*). (**b**) Main effects. Venn diagram of mtDEGs for the OCM effect (−OCM vs. +OCM, pooled across gain) and the Gain effect (CON vs. RES, pooled across OCM). The overlap indicates genes influenced by both main effects; callouts provide representative examples (e.g., *ACSM1*, *FABP4*). Fold-change convention: log_2_(Group2/Group1) for the stated contrast.

In contrast to the liver, fetal muscle tissues exhibited a greater number of mtDEGs across different pairwise comparisons and main effects of OCM and Gain (FDR ≤ 0.10; Table 5, Table 6, Table 7, Table 8 and Table 9). Notably, the majority of mtDEGs were downregulated in the CON + OCM, RES + OCM, and RES − OCM groups compared to the CON − OCM group, with the exception of *RPL12*, which was consistently upregulated (Table 5, Table 6, Table 7, Table 8 and Table 9). No mtDEGs were detected in the pairwise comparisons of RES − OCM vs. CON + OCM, CON + OCM vs. RES + OCM, and RES − OCM vs. RES + OCM (FDR ≥ 0.10). Approximately one-third of the identified mtDEGs were shared across different treatment analyses, and many of these genes overlapped between pairwise comparisons and main effects (Figure 2). Commonly identified genes across pairwise comparisons included *ACSM1*, *AGPAT2*, *FABP4*, *FASN*, *PCK2*, and *RPL12* (Figure 2a). Similarly, for the main effects of OCM and Gain, overlapping genes included *ACSM1*, *FABP4*, *PCK1*, and *PCK2* (Figure 2b). Complete mitochondria-related gene expression tables for all fetal muscle contrasts are provided in Supplementary File S2.

### 3.5. Functional and Pathway Analysis of mtDEGs in Fetal Muscle

**Figure 3:**
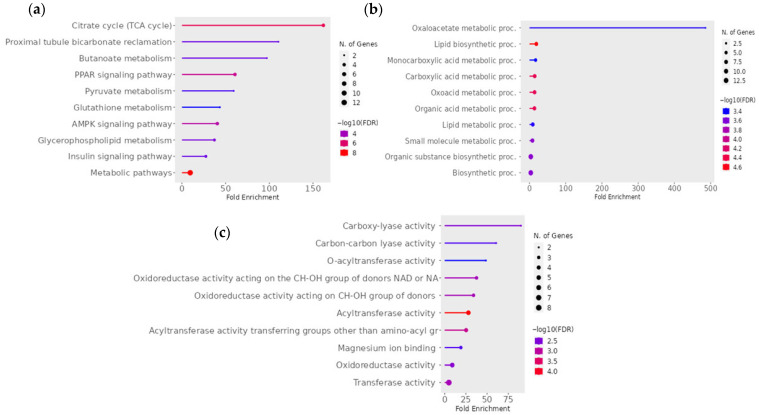
Significant (**a**) KEGG pathway terms, (**b**) GO Biological Process, and (**c**) GO Molecular Function associated with the overlap downregulated gene targets of mRNAs for the pairwise comparison between CON − OCM vs. CON + OCM for fetal muscle. The size and color of the dots represent the number of genes and the range of statistical significance, respectively. The red color indicates higher −log10(FDR) values, followed by pink, purple and blue colors. The *y*-axis represents the GO and KEGG terms, and the *x*-axis represents the fold enrichment. The *p* values were corrected for multiple tests using the false discovery rate (FDR) ≤ 0.05. The enrichment tests were performed using ShinyGO tools (http://bioinformatics.sdstate.edu/go/; accessed on 2 December 2024).

**Figure 4:**
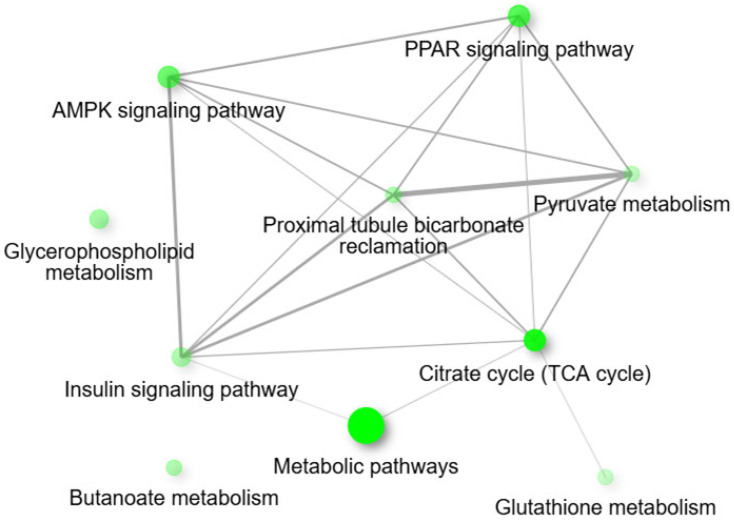
Interactive plot showing the relationship between enriched pathways (at an Enrichment FDR ≤ 0.05) for the pairwise comparison between CON − OCM vs. CON + OCM for fetal muscle. Two pathways (nodes) are connected if they share 20% or more genes. Darker nodes are more significantly enriched gene sets. Bigger nodes represent larger gene sets. Thicker edges represent more overlapped genes. The network constructions were performed using ShinyGO tools (http://bioinformatics.sdstate.edu/go/; accessed on 2 December 2024).

For the pairwise comparison of CON − OCM vs. CON + OCM (Figure 3) in fetal muscle, KEGG pathway analysis revealed enrichment in metabolic pathways such as the citrate cycle (TCA cycle), proximal tubule bicarbonate reclamation, butanoate metabolism, PPAR signaling pathway, and pyruvate metabolism (FDR ≤ 0.05). An interactive pathway network analysis (Figure 4) further illustrated the interconnections between these enriched pathways. The citrate cycle (TCA cycle) emerged as a central hub, linking with key metabolic and signaling pathways such as pyruvate metabolism, PPAR signaling pathway, and proximal tubule bicarbonate reclamation. Notably, pathways like the AMPK signaling pathway and insulin signaling pathway also exhibited associations. Gene Ontology (GO) enrichment analysis further identified several highly enriched biological processes, including oxaloacetate metabolism, lipid biosynthesis, monocarboxylic, carboxylic, and oxoacid metabolism (FDR ≤ 0.05). GO molecular function analysis indicated significant enrichment in activities such as carboxy-lyase activity, carbon-carbon lyase activity, o-acyltransferase activity, and oxidoreductase activity acting on the CH-OH group of donors with NAD or NADP as acceptors (FDR ≤ 0.05).

**Figure 5:**
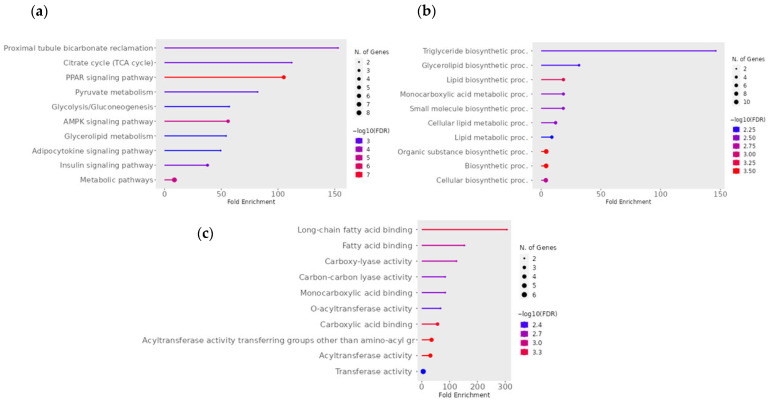
Significant (**a**) KEGG pathway terms, (**b**) GO Biological Process, and (**c**) GO Molecular Function associated with the overlap downregulated gene targets of mRNAs for the pairwise comparison between CON − OCM vs. RES + OCM for fetal muscle. The size and color of the dots represent the number of genes and the range of statistical significance, respectively. The red color indicates higher −log10(FDR) values, followed by pink, purple and blue colors. The *y*-axis represents the GO and KEGG terms, and the *x*-axis represents the fold enrichment. The *p* values were corrected for multiple tests using the false discovery rate (FDR) ≤ 0.05. The enrichment tests were performed using ShinyGO tools (http://bioinformatics.sdstate.edu/go/; accessed on 2 December 2024).

**Figure 6:**
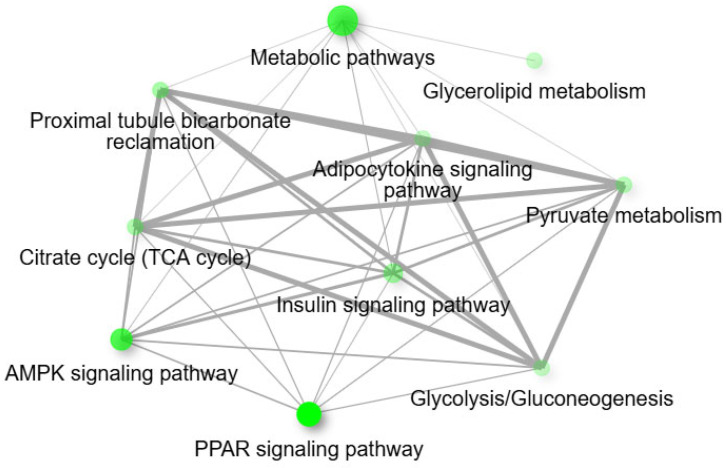
Interactive plot showing the relationship between enriched pathways (at an Enrichment FDR ≤ 0.05) for the pairwise comparison between CON − OCM vs. RES + OCM for fetal muscle. Two pathways (nodes) are connected if they share 20% or more genes. Darker nodes are more significantly enriched gene sets. Bigger nodes represent larger gene sets. Thicker edges represent more overlapped genes. The network constructions were performed using ShinyGO tools (http://bioinformatics.sdstate.edu/go/; accessed on 2 December 2024).

For the CON − OCM vs. RES + OCM comparison, KEGG pathway analysis identified significant enrichment in pathways related to mitochondrial energy metabolism and cellular signaling (Figure 5a). Among the most enriched pathways were the citrate cycle (TCA cycle), proximal tubule bicarbonate reclamation, pyruvate metabolism, glycolysis/gluconeogenesis and PPAR signaling pathway, with additional contributions from pathways such as AMPK signaling, insulin signaling, and glycerophospholipid metabolism (FDR ≤ 0.05). An interactive pathway network visualization (Figure 6) further highlighted the interconnected nature of these pathways. Pathways like citrate cycle (TCA cycle), pyruvate metabolism and glycolysis/gluconeogenesis were strongly linked to key regulators such as the PPAR signaling pathway, AMPK signaling pathway, and insulin signaling pathway (FDR ≤ 0.05). The network also revealed shared gene sets among the enriched pathways, with metabolic pathways acting as a broader umbrella term for these interrelated processes. GO analysis revealed highly enriched biological processes and molecular functions associated with metabolic regulation in fetal muscle (Figure 5b,c). Significant biological processes included triglyceride biosynthetic process, monocarboxylic acid metabolism, and lipid biosynthesis, while molecular functions were enriched for activities such as long-chain fatty acid binding, fatty acid binding, carboxy-lyase activity, carbon-carbon lyase activity, monocarboxylic acid binding (FDR ≤ 0.05).

**Figure 7:**
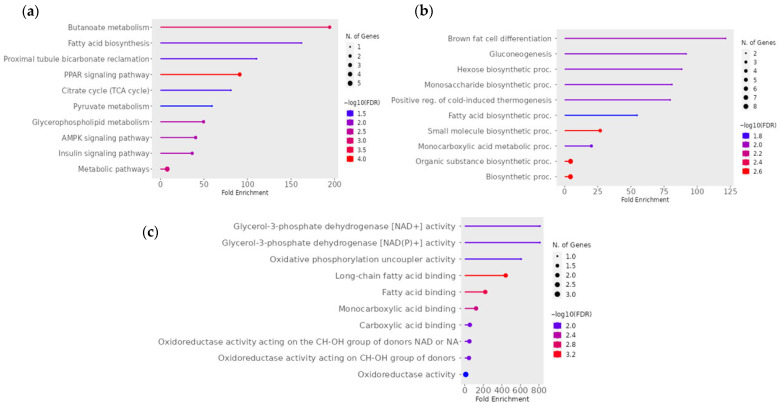
Significant (**a**) KEGG pathway terms, (**b**) GO Biological Process, and (**c**) GO Molecular Function associated with the overlap downregulated gene targets of mRNAs for the pairwise comparison between CON − OCM vs. RES − OCM for fetal muscle. The size and color of the dots represent the number of genes and the range of statistical significance, respectively. The red color indicates higher −log10(FDR) values, followed by pink, purple and blue colors. The *y*-axis represents the GO and KEGG terms, and the *x*-axis represents the fold enrichment. The *p* values were corrected for multiple tests using the false discovery rate (FDR) ≤ 0.05. The enrichment tests were performed using ShinyGO tools (http://bioinformatics.sdstate.edu/go/; accessed on 2 December 2024).

**Figure 8:**
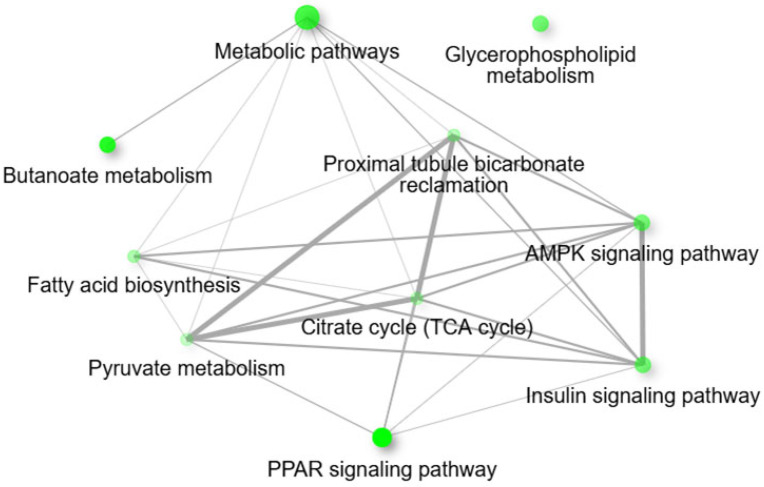
Interactive plot showing the relationship between enriched pathways (at an Enrichment FDR ≤ 0.05) for the pairwise comparison between CON − OCM vs. RES − OCM for fetal muscle. Two pathways (nodes) are connected if they share 20% or more genes. Darker nodes are more significantly enriched gene sets. Bigger nodes represent larger gene sets. Thicker edges represent more overlapped genes. The network constructions were performed using ShinyGO tools (http://bioinformatics.sdstate.edu/go/; accessed on 2 December 2024).

The KEGG pathway analysis for the pairwise comparison between CON − OCM and RES − OCM revealed significant enrichment in pathways associated with energy metabolism, lipid biosynthesis, and cellular signaling processes (Figure 7a). Key enriched pathways included butanoate metabolism, fatty acid synthesis, proximal tubule bicarbonate reclamation, PPAR signaling pathway, the citrate cycle (TCA cycle), pyruvate metabolism, and along with regulatory pathways such as AMPK signaling and insulin signaling (FDR ≤ 0.05). An interactive network of enriched pathways (Figure 8) emphasized the interconnected nature of metabolic and signaling pathways. The insulin signaling pathway, citrate cycle (TCA cycle), AMPK signaling pathway, proximal tubule bicarbonate reclamation, and pyruvate metabolism were closely linked (FDR ≤ 0.05). This network highlights the coordinated shifts in mitochondrial energy regulation, signaling, and metabolic adaptation. GO enrichment analysis highlighted a range of biological processes and molecular functions relevant to mitochondrial metabolism and energy regulation (Figure 7b,c). Significant biological processes included brown fat cell differentiation, gluconeogenesis, hexose and monosaccharide biosynthetic processes, and cold-induced thermogenesis, while molecular functions were enriched for activities such as glycerol 3-phosphate dehydrogenase [NAD+] and [NAD(P)+] activity, oxidative phosphorylation uncoupler activity, long chain fatty acid binding, and fatty acid binding (FDR ≤ 0.05).

For the main effect of OCM supplementation in fetal muscle, KEGG pathway analysis revealed significant enrichment of pathways associated with mitochondrial metabolism, lipid biosynthesis, and energy regulation (Supplementary Figure S1a). Key enriched pathways included the citrate cycle (TCA cycle), proximal tubule bicarbonate reclamation, pyruvate metabolism, and PPAR signaling pathway, as well as other metabolic pathways such as glycolysis/gluconeogenesis, AMPK signaling pathway, glycerophospholipid metabolism, and adipocytokine signaling pathway (FDR ≤ 0.05). An interactive network of enriched pathways (Supplementary Figure S3) highlighted strong interconnections between pathways, particularly the citrate cycle, pyruvate metabolism, adipocytokine signaling pathway, proximal tubule bicarbonate reclamation, insulin signaling pathway, glycolysis/gluconeogenesis, and AMPK signaling pathway (FDR ≤ 0.05). GO enrichment analysis identified key biological processes and molecular functions (Supplementary Figure S1b,c). Enriched biological processes included triglyceride biosynthetic process, neutral lipid biosynthetic process, monocarboxylic acid metabolism, and lipid and glycerolipid biosynthetic processes (FDR ≤ 0.05). Molecular functions were enriched for activities such as transketolase activity, glycerol-3-phosphate O-acyltransferase activity, transketolase activity, carboxy-lyase activity and carbon-carbon lyase activity (FDR ≤ 0.05).

KEGG pathway analysis for the main effects of gain (CON vs. RES) identified several enriched pathways associated with mitochondrial metabolism, energy regulation, and signaling (Supplementary Figure S2a). The most enriched pathways included the citrate cycle (TCA cycle), PPAR signaling pathway, pyruvate metabolism, proximal tubule bicarbonate reclamation, and glycolysis/gluconeogenesis (FDR ≤ 0.05). Regulatory pathways such as AMPK signaling, FoxO signaling, insulin resistance, and adipocytokine signaling pathway were also enriched. An interactive network plot (Supplementary Figure S4) illustrated the relationships between enriched pathways. All pathways, including citrate cycle (TCA cycle), PPAR signaling pathway, AMPK signaling, pyruvate metabolism, and glycolysis/gluconeogenesis, were highly interconnected (FDR ≤ 0.05). The thicker edges and denser network structure indicate a high degree of shared gene sets and functional overlaps among these pathways. GO analysis revealed several enriched biological processes and molecular functions related to mitochondrial metabolism and thermogenesis (Supplementary Figure S2b,c). Biological processes included brown fat cell differentiation, gluconeogenesis, hexose biosynthetic process, monosaccharide biosynthetic process, cold-induced thermogenesis, adaptive thermogenesis (FDR ≤ 0.05). Molecular functions were enriched for activities such as oxidative phosphorylation uncoupler activity, carboxy-lyase activity, long-chain fatty acid binding, and fatty acid binding (FDR ≤ 0.05).

## 4. Discussion

We hypothesized that restricted maternal nutrition would impair mitochondrial functionality and metabolic programming, and that OCM supplementation would mitigate these effects in a tissue-dependent manner, reflecting the distinct developmental roles of the fetal liver and muscle. The objective of this study was to evaluate how maternal nutrition and OCM supplementation during early gestation influence mitochondrial function, mtDNA copy number, and mitochondria-related gene expression in fetal liver and muscle tissues at day 161 of gestation in beef heifers. Despite the growing recognition of the role of OCMs in developmental programming [11,34], the underlying mechanisms by which maternal nutrition and OCM supplementation influence fetal mitochondrial metabolism remain poorly understood, particularly in livestock. To address this, we utilized a combination of molecular techniques, including seahorse analysis for mitochondrial respiration, quantitative PCR for mtDNA copy number determination, and transcriptomic profiling to explore mitochondria-related gene patterns. Our findings reveal that, in first-calf heifers, maternal nutrition and OCM supplementation during early gestation induce lasting, tissue-specific programming effects on fetal mitochondrial function and gene expression, highlighting distinct adaptive metabolic strategies in different fetal tissues observable at day 161 of gestation. It is possible that similar nutritional and OCM supplementation strategies in mature multiparous cows could yield different outcomes due to greater nutrient reserves and altered metabolic priorities compared with first-calf heifers [35]. Additionally, fetal sex may influence programming responses, as male and female fetuses can differ in growth trajectories, placental function, and metabolic adaptations, which could modify the observed tissue-specific mitochondrial responses [36]. In the fetal liver, OCM supplementation influenced mitochondrial respiration rates and mtDNA copy number, suggesting its role in supporting hepatic energy metabolism and oxidative capacity under varying maternal nutritional conditions. These findings align with the liver’s critical role in nutrient processing and metabolic regulation during fetal development [37]. Conversely, fetal muscle showed an increased mtDNA copy number with restricted gain and a broad downregulation of mitochondria-related genes (with or without OCM) but no changes in respiration, consistent with transcriptional reprogramming with potential effects on later metabolic function (e.g., fuel use and growth efficiency). These results emphasize the importance of tissue-specific mitochondrial adaptations in developmental programming and highlight the critical role of maternal nutrition during early gestation in shaping fetal growth and metabolic potential.

Previous findings from our group revealed that OCM supplementation, administered to pregnant heifers from breeding until day 63 of gestation, did not influence fetal mitochondrial respiration in liver and muscle tissues at day 63 [25]; however, the significant interaction effects of Gain × OCM observed for mitochondrial respiration in fetal liver at day 161 of gestation in the current study suggest that the developmental stage of fetal tissues as well as the programmed memory from previous nutritional modifications plays a role in their metabolic responsiveness. At day 63 of gestation, fetal organs are still in their early developmental stages, characterized by relatively low metabolic demand. Furthermore, the day 63 study revealed an interaction effect of weight gain rate and OCM supplementation on the allometric growth of vital fetal tissues. Specifically, the allometric growth of fetal cardiac and left longissimus dorsi tissues, as well as brain hemispheres, was increased in the CON + OCM group compared to the CON − OCM group, indicating that OCM supplementation may influence tissue-selective growth patterns even in early gestation [38]. By day 161, the fetal liver undergoes substantial functional and structural maturation, including enhanced mitochondrial biogenesis, increased enzymatic activity, and greater metabolic capacity, making it more responsive to maternal nutritional interventions [7,39]. The liver’s role as a central hub for nutrient processing and metabolic regulation becomes more pronounced later in gestation [40], likely increasing its sensitivity to maternal dietary inputs and OCM supplementation at this stage. Supporting this, Crouse et al., (2022) demonstrated that supplementation with epigenetic modifiers, including methionine, choline, folate, and vitamin B_12_, enhanced mitochondrial maximal respiration and reserve capacity in bovine embryonic fibroblast cells, resulting in improved cell growth in a glucose- and dose-dependent manner [41]. The study attributed these alterations to changes in DNA methylation, which regulated genes involved in growth and energy metabolism. While this study was conducted in bovine cells, it underscores the critical role of one-carbon metabolites in enhancing mitochondrial function.

In this study, mtDNA copy number was increased in the RES and +OCM groups compared to the CON and −OCM groups, respectively, for fetal liver. In fetal muscle, mtDNA copy number was also higher in the RES group compared to the CON group, but no differences were observed for OCM supplementation. Conversely, mitochondrial respiration in fetal liver showed significant Gain × OCM interaction effects, indicating that maternal nutrition and OCM supplementation together influenced mitochondrial functionality in the liver. However, no significant differences in mitochondrial respiration were observed for fetal muscle across treatment groups. These results suggest a tissue-specific response, where the liver demonstrated heightened sensitivity to maternal nutrition and OCM supplementation, while muscle responded differently, with changes in mtDNA copy number and transcriptional regulation but no alteration in respiratory activity at this developmental stage. To understand these findings, it is important to consider the biological role of mtDNA. The mammalian mtDNA is a circular double-stranded DNA molecule present in multiple copies per cell [42]. Mitochondria DNA copy number reflects both the number of mitochondria per cell and the number of mitochondrial genomes per mitochondrion. As such, mtDNA copy number is often used as an indirect biomarker of mitochondrial function. Although an increase in mtDNA copy number is generally associated with enhanced mitochondrial biogenesis [43], it does not always correlate directly with mitochondrial respiration. This disconnect was evident in fetal muscle, where significant differences in mtDNA copy number for Gain were not accompanied by corresponding changes in mitochondrial respiration, suggesting that mitochondrial functionality depends on additional factors, such as mitochondrial efficiency or enzymatic activity [44]. Notably, previous studies have shown that altered mtDNA copy number can serve as a biomarker for fetal development and pregnancy complications. For instance, reduced mtDNA copy number in peripheral blood during early pregnancy has been linked to preeclampsia with intrauterine growth restriction (PE-IUGR), likely reflecting mitochondrial dysfunction and oxidative stress in circulating nucleated cells [45]. While low mtDNA levels in PE-IUGR pregnancies indicate mitochondrial dysfunction, the elevated mtDNA copy number observed in our study may represent an adaptive response to maternal nutrition, potentially supporting improved fetal metabolic capacity. Taken together, the liver profile (↑ state 3; ↑ mtDNA) is consistent with enhanced oxidative capacity and nutrient processing at mid-gestation, whereas the muscle profile (no respiration change with coordinated transcriptomic downregulation) suggests a substrate-conserving state during myofiber maturation that could influence postnatal fuel preference and growth efficiency.

A greater number of mtDEGs were identified in fetal muscle (19 genes) compared to fetal liver (2 genes). This disparity likely reflects the different metabolic demands and developmental priorities of muscle and liver during gestation. The liver, as a central organ for nutrient metabolism and processing, may exhibit tighter regulation of mitochondria-related gene expression, whereas the muscle, undergoing rapid structural growth and differentiation [46], may show broader transcriptional changes in response to maternal nutritional interventions. Among the most commonly identified and downregulated mtDEGs were *ACSM1*, *FABP4*, and *PCK*, which are critical regulators of metabolic pathways central to energy homeostasis and tissue development. *ACSM1* (Acyl-CoA synthetase medium-chain family member 1) facilitates the activation of medium-chain fatty acids for β-oxidation [47], a key process for mitochondrial energy production and fatty acid metabolism. Its downregulation may indicate a shift away from fatty-acid activation/oxidation and tighter ATP coupling in developing muscle. Similarly, *FABP4* (Fatty acid-binding protein 4), which is involved in intracellular lipid transport and storage [48], was also downregulated, suggesting a decrease in lipid mobilization and utilization. This may point to a shift in the muscle’s metabolic priorities, favoring structural development over lipid metabolism under these conditions. *PCK* (Phosphoenolpyruvate carboxykinase), a key enzyme in gluconeogenesis [49], was also downregulated. Although fetal gluconeogenesis is typically minimal during mid-gestation, this downregulation may reflect anticipatory regulation of carbon flux rather than reduced active glucose production. Collectively, these muscle gene changes indicate transcriptional reprogramming toward substrate conservation at day 161, with potential consequences for postnatal feed efficiency, body composition, and growth rate.

The coordinated downregulation of *ACSM1*, *FABP4*, and *PCK* in fetal muscle in response to restricted maternal gain and OCM supplementation suggests that these maternal dietary interventions influence key metabolic pathways by attenuating fatty acid oxidation, lipid transport, and gluconeogenesis. These transcriptional changes likely reflect a shift in substrate utilization during late gestation, a period when myofiber hypertrophy and tissue maturation dominate muscle development. Importantly, as all fetuses in this study were female, these adaptations may represent a strategy to support long-term maintenance efficiency and reproductive longevity. By reallocating energy toward structural development and cellular differentiation, fetal muscle may be metabolically primed for future growth and function. These findings underscore the potential of maternal diet and OCM supplementation to induce lasting changes in fetal muscle bioenergetics and developmental programming.

Previously, a study demonstrated that moderate maternal nutrient restriction (40% global nutrient restriction) during the first 50 days of gestation in beef heifers alters transcript abundance of genes associated with tissue metabolism, accretion, and function in fetal liver, muscle, and cerebrum [50]. Their findings showed that most differentially expressed genes in the RES group were upregulated, suggesting that moderate nutrient restriction during early gestation triggers compensatory mechanisms to support tissue metabolism and development. In contrast, our study focused on nutrient restriction characterized by actual weight loss (−0.23 kg/day), which likely represents a more profound metabolic challenge to the developing fetus. This distinction may explain our findings of predominantly downregulated mtDEGs in fetal muscle in response to nutrient restriction, highlighting that true nutrient restriction can have a greater suppressive effect on gene expression patterns in metabolically active tissues. These results may underscore the differential impact of varying levels of nutrient restriction on fetal metabolic programming and developmentally distinct gene expression during critical windows of development.

The most upregulated gene in both fetal liver and muscle tissues was *RPL12*, based on its large fold-change magnitude and statistical significance, with no evidence of downregulation in any comparisons. Expression of this gene was 1500 to 2500-fold greater in the CON + OCM, RES + OCM, and RES − OCM groups compared to the CON − OCM group. The consistent upregulation of *RPL12* across tissues highlights its potential as a critical mediator of cellular and metabolic responses to maternal nutrition and OCM supplementation. *RPL12* is a ribosomal protein involved in the assembly and function of ribosomes [51], which are essential for protein synthesis. Its upregulation likely reflects an increased demand for protein translation in fetal tissues, potentially driven by enhanced cellular growth and metabolic activity during gestation. Both liver and muscle tissues are metabolically and structurally active during fetal development. Elevated *RPL12* expression in these tissues could support vital processes, such as biosynthesis (e.g., amino acid metabolism and protein translation) and structural growth, rather than implying active fetal gluconeogenesis at this stage. The lower expression observed in the CON − OCM group suggests that maternal OCM supplementation and nutritional plane positively modulate *RPL12* expression, enhancing ribosomal activity and overall protein synthesis capacity. These transcriptional patterns align with observed mitochondrial respiration data in liver tissue, where Gain × OCM interaction effects were detected. Specifically, state 3 respiration (indicative of active ATP production) and state 4o respiration (reflecting proton leak) were highest in the CON + OCM group, suggesting elevated mitochondrial oxidative activity. However, the simultaneous increase in proton leak may indicate a trade-off, where enhanced respiration is accompanied by reduced coupling efficiency. In contrast, the RES + OCM group exhibited the lowest state 4o respiration, potentially reflecting improved mitochondrial efficiency with more oxygen consumption directed toward ATP production rather than dissipated as heat. Although RCR did not reach statistical significance, the trend toward greater RCR in +OCM groups supports this interpretation. The upregulation of *RPL12*, coupled with increased mitochondrial respiration and mtDNA copy number in the liver, may be indicative of the tissue’s high metabolic adaptability to maternal dietary interventions. Conversely, the lack of similar effects in muscle suggests that ribosomal and mitochondrial adaptations are prioritized differently across tissues, with liver being more metabolically responsive while structural development is emphasized more in muscle.

In fetal liver, the observed functional responses, such as elevated state 3 respiration in response to CON + OCM and increased mitochondrial biogenesis (mtDNA copy number) in response to both restricted gain and OCM supplementation, occurred with minimal mitochondria-related gene expression changes and therefore point to regulation primarily via post-translational mechanisms. Indeed, it has been suggested that mitochondrial functionality is regulated by post-translational modifications, including phosphorylation, acetylation, succinylation, and redox modifications, that respond rapidly to environmental cues and nutrient availability independently of transcriptomic alterations [52]. Conversely, fetal muscle displayed minimal mitochondrial respiratory changes at day 161 but showed greater transcriptional regulation, predominantly characterized by the downregulation of mitochondrial-related genes involved in fatty acid metabolism, lipid transport, and substrate utilization pathways. These transcriptomic shifts, although not immediately coupled with functional mitochondrial changes, may indicate metabolic reprogramming that could manifest functionally at later developmental stages or postnatally [53], aligning with evolving metabolic demands and tissue-specific growth priorities. Thus, the mid-gestation transcriptional changes in fetal muscle could represent anticipatory metabolic adaptations. These results highlight the importance of maternal nutrition in modulating fetal metabolic programming and suggest that OCM supplementation may promote mitochondrial adaptations and transcriptional shifts that support metabolic development during critical periods of gestation. Together, these findings support a model in which liver mounts rapid functional adjustments through post-translational control, whereas muscle engages a transcriptomic program whose physiological consequences may emerge postnatally.

While the study highlights significant changes in mitochondrial function and mitochondria-related gene expression, we did not evaluate mitochondrial morphology in fetal liver and muscle. Morphological assessments (e.g., transmission electron microscopy or high-resolution confocal imaging) would clarify whether the observed molecular adaptations are accompanied by changes in mitochondrial number, size, or cristae architecture. In addition, protein-level measurements and post-translational modifications (e.g., phosphorylation, acetylation, succinylation) of key mitochondrial enzymes were not quantified; thus, inferences about post-translational regulation are indirect. Additionally, all fetuses were female and dams were first-calf heifers; therefore, generalizability to male fetuses and multiparous cows remains uncertain. Finally, outcomes were evaluated at a single mid-gestation time point (day 161) and no postnatal follow-up was performed. Future studies should incorporate mitochondrial morphology, proteomics/PTM profiling and longitudinal postnatal phenotypes to resolve mechanisms and functional consequences.

## 5. Conclusions

This study demonstrates that maternal nutrition and OCM supplementation during early gestation (day 0 to 63) results in developmentally distinct mitochondrial adaptations in fetal liver and muscle at mid-gestation (day 161). Fetal liver exhibited increased mitochondrial respiration (in response to CON + OCM) and mtDNA copy number (in response to restricted gain and OCM supplementation), highlighting its central role in nutrient metabolism and metabolic regulation during development. These changes occurred with minimal differential expression of mitochondria-related genes, suggesting metabolic regulation potentially mediated by post-translational mechanisms. In contrast, fetal muscle showed minimal changes in mitochondrial respiration and mtDNA copy number but exhibited significant downregulation of mitochondria-related genes in response to restricted maternal gain and OCM supplementation, particularly in pathways related to fatty acid metabolism, lipid transport, and gluconeogenesis. These findings may reflect anticipatory or delayed transcriptional reprogramming that prioritizes structural development over immediate metabolic flexibility. These mid-gestation findings reflect programmed effects originating from nutritional interventions applied through day 63 of gestation. This suggests that early gestational programming can induce metabolic adaptations that persist beyond the treatment period and highlights the potential of early gestation as a critical window for influencing fetal metabolic programming. However, whether these adaptations persist through late gestation or influence calf health and productivity postnatally remains unknown. Future investigations should assess whether these changes continue through late gestation and into postnatal life. Further research should also aim to elucidate the epigenetic and molecular mechanisms underlying these functionally distinct responses to maternal nutrition and OCM supplementation. Such insights could inform the development of targeted nutritional strategies to enhance reproductive efficiency and optimize offspring growth, metabolic health, and lifetime productivity in livestock systems.

## Figures and Tables

**Table 1:** Ingredient and nutrient composition of heifer diets (dry matter basis); modified from Syring et al. [28].

**Ingredient, % of Dietary DM**	
Ground corn	27.0
Corn silage	9.0
Alfalfa	15.0
Alfalfa/grass hay	45.0
Top-dressed supplement ^1^	4.0
**Average Estimated Dietary Composition**	
Dry matter, %	83.00
Crude protein, %	13.50
Dietary metabolizable energy, Mcal/kg ^2^	2.50
Dietary net energy of maintenance, Mcal/kg ^2^	2.00
Dietary net energy of gain, Mcal/kg ^2^	1.00
**Supplement Ingredients on a g/d Basis**	
Trouw dairy VTM w/Optimins ^3^	10.0
Rumen protected methionine ^4^	7.5
Rumen protected choline ^5^	44.5
**Chemical Composition of Trouw VTM Premix ^6^**	
Ca, %	10.00 to 12.00
Mg, %	5.00
K, %	5.00
Co, mg/kg	180.00
Cu, mg/kg	5100.00
I, mg/k	375.00
Fe, %	1.00
Mg, %	3.00
Se, mg/kg	132.00
Zn, %	3.00
Vitamin A, IU/kg	2,755,775.00
Vitamin D_3_, IU/kg	771,617.00
Vitamin E, IU/kg	13,228.00

^1^ Top-dressed supplement delivered using a fine ground corn carrier. For −OCM heifers, supplement comprising Trouw dairy VTM w/Optimins mix and fine ground corn carrier. For +OCM heifers, supplement comprising Trouw dairy VTM w/Optimins mix, rumen protected methionine, rumen protected choline, and fine ground corn carrier. ^2^ Calculated values from the NRC (2016). ^3^ Trouw dairy VTM w/Optimins, Trouw Nutrition USA, Highland, IL, USA. ^4^ Smartamine, Adisseo, China. ^5^ ReaShure, Balchem Inc., New Hampton, NY, USA. ^6^ Trouw VTM premix fed to meet or exceed vitamin and mineral requirements.

**Table 2:** Oxygen consumption rates and respiratory control ratios in isolated mitochondria from fetal liver collected at day 161 of gestation. Maternal treatments were administered from breeding (day 0) through day 63 of gestation (control gain vs. restricted gain; ±one-carbon metabolite supplementation).

	Restricted		Control		SEM	*p* value		
	−OCM	+OCM	−OCM	+OCM		Gain	OCM	Gain × OCM
**State 3 respiration**	586 ^ab^	482 ^a^	437 ^a^	646 ^b^	52.2	0.88	0.30	0.004
**State 4o respiration**	72.9 ^b^	57.2 ^a^	64.6 ^ab^	78.1 ^ab^	7.07	0.87	0.35	0.04
* RCR	8.04	8.43	6.76	8.27	0.99	0.85	0.08	0.37

Different superscript letters within a row indicate significant differences (*p* < 0.05). * RCR (respiratory control ratio) = State 3 ÷ State 4o. State 3 and State 4o respiration are expressed as picomoles O_2_ consumed per μg mitochondrial protein per minute. Abbreviations: +OCM = one-carbon metabolite supplementation; −OCM = no supplementation; SEM = standard error of the mean.

**Table 3:** Oxygen consumption rates and respiratory control ratio in isolated mitochondria from fetal muscle samples using coupling assay.

	Restricted		Control		SEM	*p* value		
	−OCM	+OCM	−OCM	+OCM		Gain	OCM	Gain × OCM
**State 3 respiration**	608	582	585	714	133.6	0.66	0.68	0.54
**State 4o respiration**	83.8	85.5	120	96.0	33.37	0.46	0.73	0.68
* RCR	7.25	6.81	4.88	7.44	1.259	0.61	0.99	0.76

* RCR (respiratory control ratio) = State 3 ÷ State 4o. State 3 and State 4o respiration are expressed as picomoles O_2_ consumed per μg mitochondrial protein per minute. Abbreviations: +OCM = one-carbon metabolite supplementation; −OCM = no supplementation; SEM = standard error of the mean.

**Table 4:** Mitochondria-related differentially expressed genes (mtDEGs) in fetal liver at day 161 of gestation: pairwise contrasts among maternal treatments.

Treatment Group	Name	Ensembl ID	Fold Change	FDR *p* Value	*p* Value	Biotype
CON − OCM vs. CON + OCM	*RPL12*	ENSBTAG00000006963	2484.8215	<0.001	<0.001	protein_coding
CON − OCM vs. RES + OCM	*RPL12*	ENSBTAG00000006963	1104.8939	<0.001	<0.001	protein_coding
	*CYP3A5_2*	ENSBTAG00000053645	−8.1855	0.007	<0.001	protein_coding
RES − OCM vs. RES + OCM	*CYP3A5_2*	ENSBTAG00000053645	−8.5048	0.005	<0.001	protein_coding
CON − OCM vs. RES − OCM	*RPL12*	ENSBTAG00000006963	1535.9445	<0.001	<0.001	protein_coding

Maternal treatments were administered from breeding (day 0) through day 63 of gestation in first-calf heifers: control gain (CON; 0.45 kg/day) vs. restricted gain (RES; −0.23 kg/day) and one-carbon metabolite supplementation (+OCM; methionine, choline, folate, vitamin B_12_) vs. no supplementation (−OCM). Fetal Liver samples were collected at day 161 of gestation. Fold change is log_2_(Group2/Group1) for the stated contrast. Abbreviations: CON = control gain; RES = restricted gain; OCM = one-carbon metabolite supplementation; mtDEG = mitochondria-related differentially expressed gene; FDR = false discovery rate; Ensembl ID = Ensembl gene identifier.

**Table 5:** Mitochondria-related differentially expressed genes (mtDEGs) in fetal muscle at day 161 of gestation: CON − OCM vs. CON + OCM.

Name	Ensembl ID	Fold Change	FDR *p* Value	*p* Value	Biotype
*ACLY*	ENSBTAG00000016740	−2.8592	0.0391	0.000111	protein_coding
*ACSM1*	ENSBTAG00000001417	−130.5345	0.0001	<0.001	protein_coding
*AGPAT2*	ENSBTAG00000025161	−4.4916	0.0163	<0.001	protein_coding
*BDH1*	ENSBTAG00000000448	−4.9496	0.0509	<0.001	protein_coding
*FABP4*	ENSBTAG00000037526	−176.6854	0.0000	<0.001	protein_coding
*FASN*	ENSBTAG00000015980	−4.7550	0.0078	<0.001	protein_coding
*GLYAT*	ENSBTAG00000038323	−45.5870	0.0032	<0.001	protein_coding
*GPAM*	ENSBTAG00000011917	−5.0158	0.0177	<0.001	protein_coding
*GPD1*	ENSBTAG00000016296	−3.0421	0.0875	<0.001	protein_coding
*IDH1*	ENSBTAG00000020527	−3.3821	0.0479	<0.001	protein_coding
*LRRC39*	ENSBTAG00000015786	2.2008	0.0447	<0.001	protein_coding
*MGST1*	ENSBTAG00000008541	−4.7780	0.0600	<0.001	protein_coding
*PCK2*	ENSBTAG00000011934	−5.4512	0.0096	<0.001	protein_coding
*PCK1*	ENSBTAG00000001936	−15.8713	0.0001	<0.001	protein_coding
*PPARG*	ENSBTAG00000001333	−9.8412	0.0002	<0.001	protein_coding
*RPL12*	ENSBTAG00000006963	295.4703	0.0000	<0.001	protein_coding
*SLC16A11*	ENSBTAG00000020852	−4.6394	0.0215	<0.001	protein_coding
*TKT*	ENSBTAG00000003758	−3.1795	0.0244	<0.001	protein_coding

Maternal treatments were administered from breeding (day 0) through day 63 of gestation in first-calf heifers: control gain (CON; 0.45 kg/day) vs. restricted gain (RES; −0.23 kg/day) and one-carbon metabolite supplementation (+OCM; methionine, choline, folate, vitamin B_12_) vs. no supplementation (−OCM). Fetal muscle samples were collected at day 161 of gestation. Fold change is log_2_(Group2/Group1) for the stated contrast. Abbreviations: CON = control gain; RES = restricted gain; OCM = one-carbon metabolite supplementation; mtDEG = mitochondria-related differentially expressed gene; FDR = false discovery rate; Ensembl ID = Ensembl gene identifier.

**Table 6:** Mitochondria-related differentially expressed genes (mtDEGs) in fetal muscle at day 161 of gestation: CON − OCM vs. RES + OCM.

Name	Ensembl ID	Fold Change	FDR *p* Value	*p* Value	Biotype
*ACSM1*	ENSBTAG00000001417	−134.9288	0.0004	<0.001	protein_coding
*AGPAT2*	ENSBTAG00000025161	−4.6326	0.0172	<0.001	protein_coding
*FABP4*	ENSBTAG00000037526	−68.8895	0.0000	<0.001	protein_coding
*FASN*	ENSBTAG00000015980	−4.2455	0.0421	<0.001	protein_coding
*GLYAT*	ENSBTAG00000038323	−29.9011	0.0168	<0.001	protein_coding
*GPAM*	ENSBTAG00000011917	−4.7862	0.0413	<0.001	protein_coding
*PCK2*	ENSBTAG00000011934	−5.0033	0.0384	<0.001	protein_coding
*PCK1*	ENSBTAG00000001936	−22.7321	0.0000	<0.001	protein_coding
*PPARG*	ENSBTAG00000001333	−6.0840	0.0855	0.000226	protein_coding
*RPL12*	ENSBTAG00000006963	171.7712	0.0000	<0.001	protein_coding
*SLC16A11*	ENSBTAG00000020852	−3.7431	0.0914	0.000251	protein_coding
*TKT*	ENSBTAG00000003758	−3.2251	0.0521	0.000135	protein_coding
*UCP1*	ENSBTAG00000004647	−23.3073	0.0878	0.000237	protein_coding

Maternal treatments were administered from breeding (day 0) through day 63 of gestation in first-calf heifers: control gain (CON; 0.45 kg/day) vs. restricted gain (RES; −0.23 kg/day) and one-carbon metabolite supplementation (+OCM; methionine, choline, folate, vitamin B_12_) vs. no supplementation (−OCM). Fetal muscle samples were collected at day 161 of gestation. Fold change is log_2_(Group2/Group1) for the stated contrast. Abbreviations: CON = control gain; RES = restricted gain; OCM = one-carbon metabolite supplementation; mtDEG = mitochondria-related differentially expressed gene; FDR = false discovery rate; Ensembl ID = Ensembl gene identifier.

**Table 7:** Mitochondria-related differentially expressed genes (mtDEGs) in fetal muscle at day 161 of gestation: CON − OCM vs. RES-OCM.

Name	Ensembl ID	Fold Change	FDR *p* Value	*p* Value	Biotype
*ACSM1*	ENSBTAG00000001417	−42.8149	0.0038	<0.001	protein_coding
*AGPAT2*	ENSBTAG00000025161	−4.5288	0.0247	<0.001	protein_coding
*FABP4*	ENSBTAG00000037526	−30.3606	0.0027	<0.001	protein_coding
*BDH1*	ENSBTAG00000000448	−4.6942	0.0681	0.000165	protein_coding
*FASN*	ENSBTAG00000015980	−4.2605	0.0589	0.000131	protein_coding
*GPD1*	ENSBTAG00000016296	−3.2988	0.0954	0.000258	protein_coding
*PCK2*	ENSBTAG00000011934	−6.6713	0.0033	<0.001	protein_coding
*RPL12*	ENSBTAG00000006963	169.0700	0.0000	<0.001	protein_coding
*UCP1*	ENSBTAG00000004647	−34.6716	0.0792	0.000209	protein_coding

Maternal treatments were administered from breeding (day 0) through day 63 of gestation in first-calf heifers: control gain (CON; 0.45 kg/day) vs. restricted gain (RES; −0.23 kg/day) and one-carbon metabolite supplementation (+OCM; methionine, choline, folate, vitamin B_12_) vs. no supplementation (−OCM). Fetal muscle samples were collected at day 161 of gestation. Fold change is log_2_(Group2/Group1) for the stated contrast. Abbreviations: CON = control gain; RES = restricted gain; OCM = one-carbon metabolite supplementation; mtDEG = mitochondria-related differentially expressed gene; FDR = false discovery rate; Ensembl ID = Ensembl gene identifier.

**Table 8:** Mitochondria-related differentially expressed genes (mtDEGs) in fetal muscle at day 161 of gestation: −OCM vs. +OCM.

Name	Ensembl ID	Fold Change	FDR *p* Value	*p* Value	Biotype
*ACSM1*	ENSBTAG00000001417	−66.7699	0.0000	<0.001	protein_coding
*AGPAT2*	ENSBTAG00000025161	−2.7690	0.0459	0.000107	protein_coding
*FABP4*	ENSBTAG00000037526	−52.2625	0.0000	<0.001	protein_coding
*FASN*	ENSBTAG00000015980	−2.7656	0.0361	<0.001	protein_coding
*GLYAT*	ENSBTAG00000038323	−20.3391	0.0014	<0.001	protein_coding
*GPAM*	ENSBTAG00000011917	−3.0415	0.0391	<0.001	protein_coding
*MGST1*	ENSBTAG00000008541	−3.0183	0.0564	0.000135	protein_coding
*PCK2*	ENSBTAG00000011934	−2.9884	0.1086	0.000289	protein_coding
*PCK1*	ENSBTAG00000001936	−9.6135	0.0001	<0.001	protein_coding
*PPARG*	ENSBTAG00000001333	−4.5198	0.0089	<0.001	protein_coding
*TKT*	ENSBTAG00000003758	−2.1670	0.0707	0.000173	protein_coding

Maternal treatments were administered from breeding (day 0) through day 63 of gestation in first-calf heifers: control gain (CON; 0.45 kg/day) vs. restricted gain (RES; −0.23 kg/day) and one-carbon metabolite supplementation (+OCM; methionine, choline, folate, vitamin B_12_) vs. no supplementation (−OCM). Fetal muscle samples were collected at day 161 of gestation. Fold change is log_2_(Group2/Group1) for the stated contrast. Abbreviations: CON = control gain; RES = restricted gain; OCM = one-carbon metabolite supplementation; mtDEG = mitochondria-related differentially expressed gene; FDR = false discovery rate; Ensembl ID = Ensembl gene identifier.

**Table 9:** Mitochondria-related differentially expressed genes (mtDEGs) in fetal muscle at day 161 of gestation: CON vs. RES.

Name	Ensembl ID	Fold Change	FDR *p* Value	*p* Value	Biotype
*ACSM1*	ENSBTAG00000001417	−29.7676	0.0025	<0.001	protein_coding
*FABP4*	ENSBTAG00000037526	−19.2508	0.0025	<0.001	protein_coding
*PCK2*	ENSBTAG00000011934	−3.1443	0.0891	0.00014	protein_coding
*PCK1*	ENSBTAG00000001936	−10.4104	0.0001	<0.001	protein_coding
*UCP1*	ENSBTAG00000004647	−15.0831	0.0621	<0.001	protein_coding

Maternal treatments were administered from breeding (day 0) through day 63 of gestation in first-calf heifers: control gain (CON; 0.45 kg/day) vs. restricted gain (RES; −0.23 kg/day) and one-carbon metabolite supplementation (+OCM; methionine, choline, folate, vitamin B_12_) vs. no supplementation (−OCM). Fetal muscle samples were collected at day 161 of gestation. Fold change is log_2_(Group2/Group1) for the stated contrast. Abbreviations: CON = control gain; RES = restricted gain; OCM = one-carbon metabolite supplementation; mtDEG = mitochondria-related differentially expressed gene; FDR = false discovery rate; Ensembl ID = Ensembl gene identifier.

## Data Availability

The original contributions presented in this study are included in the article/Supplementary Materials. Further inquiries can be directed to the corresponding author.

## Supplementary Materials

The following supporting information can be downloaded at: https://www.mdpi.com/article/10.3390/ani15182689/s1, Table S1: Primers used for mitochondrial DNA copy number determination using qPCR; Figure S1: Significant (a) KEGG pathway terms, (b) GO Biological Process, and (c) GO Molecular Function associated with the overlap downregulated gene targets of mRNAs for the main effects (NoOCM vs. OCM) for fetal muscle.; Figure S2: Significant (a) KEGG pathway terms, (b) GO Biological Process, and (c) GO Molecular Function associated with the overlap downregulated gene targets of mRNAs for the main effects (CON vs. RES) for fetal muscle. Figure S3: Interactive plot showing the relationship between enriched pathways (at an Enrichment FDR ≤ 0.05) for the main effects (NoOCM vs. OCM) for fetal muscle. Figure S4: Interactive plot showing the relationship between enriched pathways (at an Enrichment FDR ≤ 0.05) for the main effects (CON vs. RES) for fetal muscle. File S1: Fetal liver mitochondria-related gene expression at day 161 across maternal gain × OCM contrasts (log_2_FC, P, FDR, CPM). File S2: Fetal muscle mitochondria-related gene expression at day 161 across maternal gain × OCM contrasts (log_2_FC, P, FDR, CPM).
