# Supplementary material for: Tissue-Specific Mitochondrial Functionality and Mitochondrial-Related Gene Profiles in Response to Maternal Nutrition and One-Carbon Metabolite Supplementation During Early Pregnancy in Heifers
Source: Animals (Basel). 2025 Sep 14;15(18):2689. doi: 10.3390/ani15182689 (PMC12466886; doi:10.3390/ani15182689)
Supplement: Supplementary file 1 [file animals-15-02689-s001.zip › Supplementary Tables and figures.pdf]

Supplementary Table 1: Primers used for mitochondrial DNA copy number determination using qPCR

**Target gene**	**Oligonucleotide sequence (5' --> 3')**
ND2 (NADH dehydrogenase subunit 2)	F: GGCCAATGAACCGTAATAAA
	R: CTGGGACTCAGAAGTGAAA
COX3 (cytochrome oxidase subunit III)	F: AGGCATTCACCCACTAAA
	R: CTATGATGGGCTCAGGTAATA
ACTB (actin B)-nuclear gene	F: AATCGTCCGTGACATCAA
	R: GAAGCTCGTAGCTCTTCTC

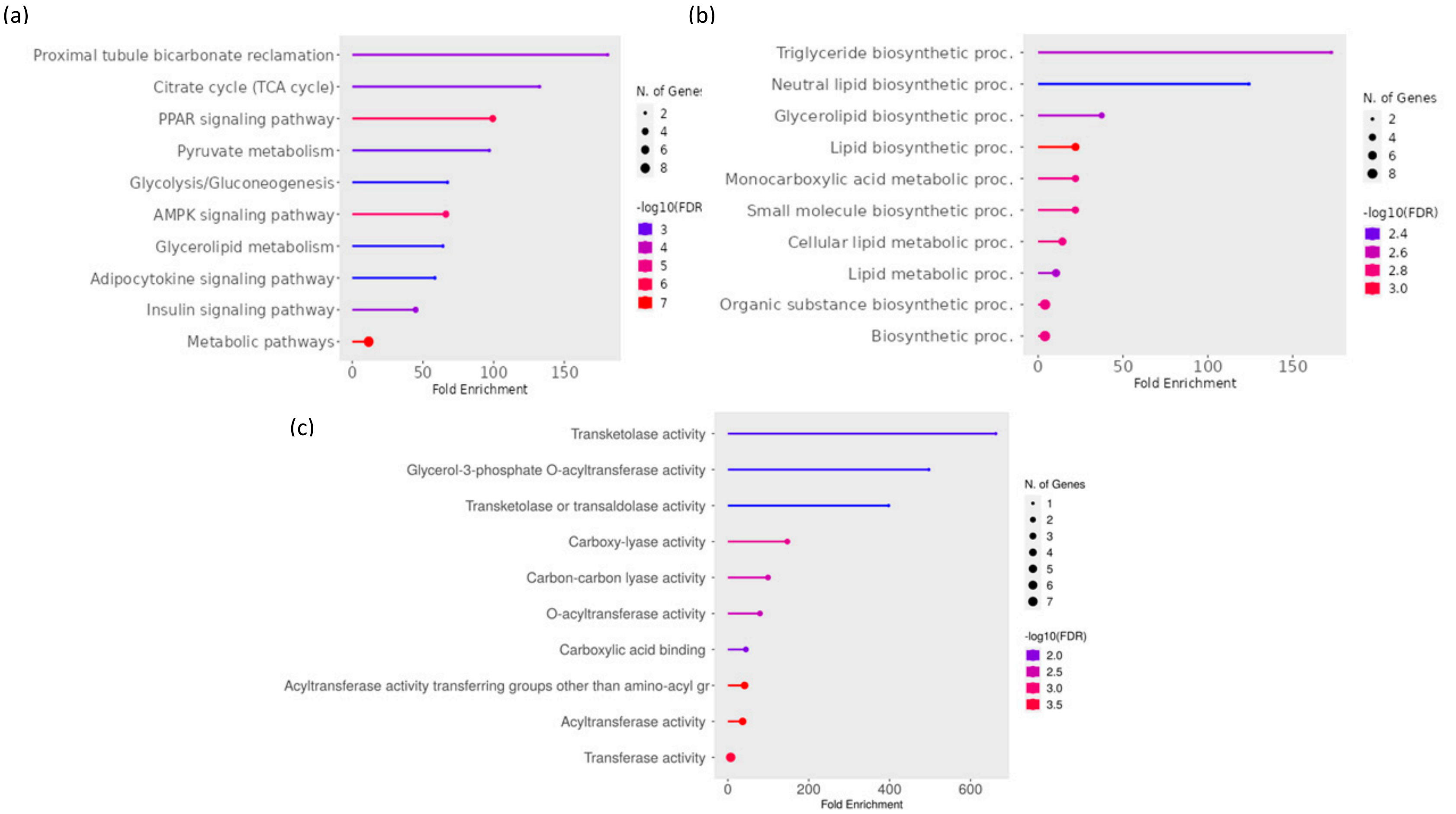

Supplementary figure 1: Significant (a) KEGG pathway terms, (b) GO Biological Process, and (c) GO Molecular Function associated with the overlap downregulated gene targets of mRNAs for the main effects (NoOCM vs OCM) for fetal muscle. The size and color of the dots represent the number of genes and the range of statistical significance, respectively. The red color indicates higher -

log10(FDR) values, followed by pink, purple and blue colors. The y-axis represents the GO and KEGG terms, and the x-axis represents the fold enrichment. The *P* values were corrected for multiple tests using the false discovery rate (FDR) ≤ 0.05. The enrichment tests were performed using ShinyGO tools (http://bioinformatics.sdstate.edu/go/)

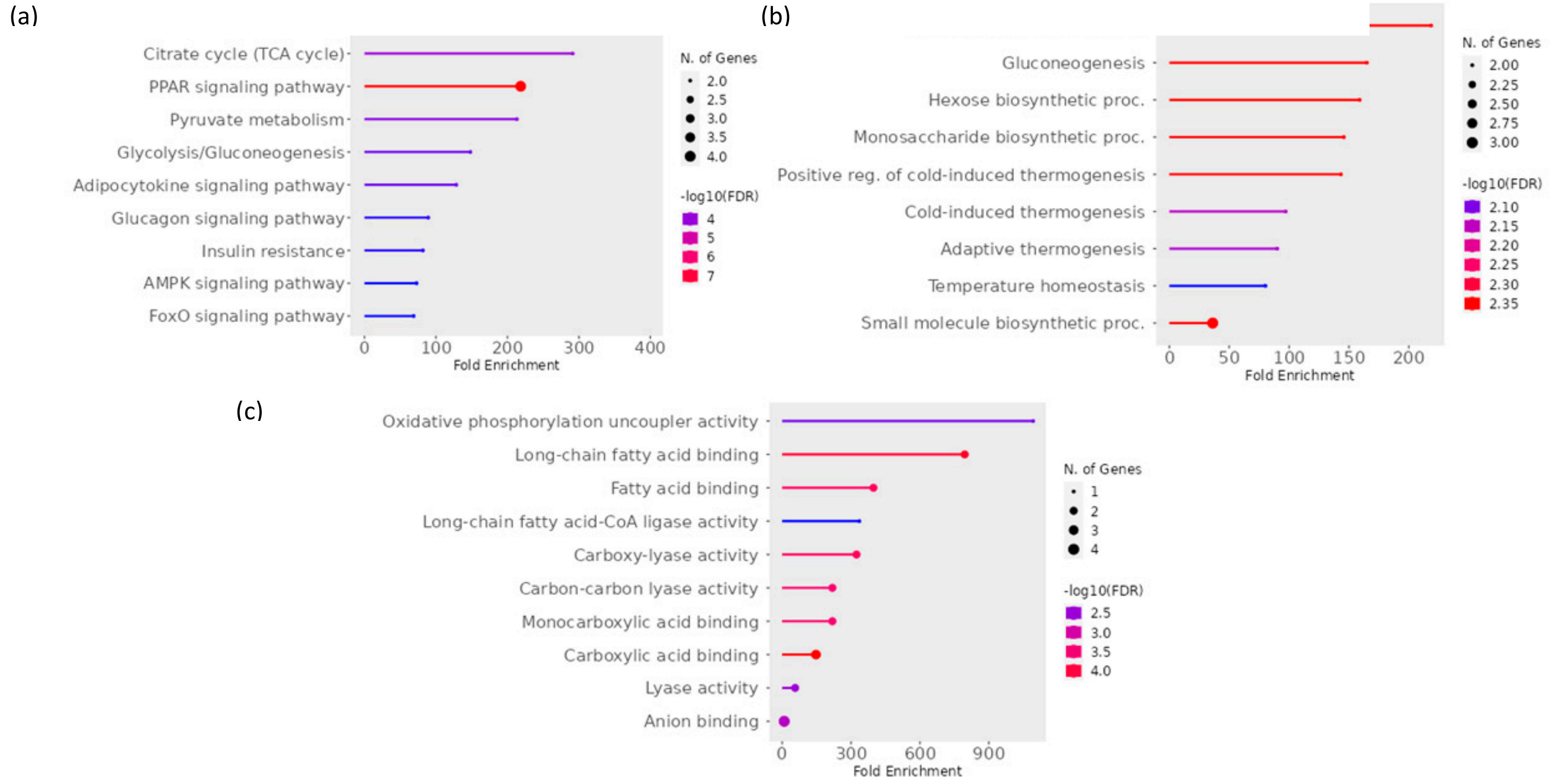

Supplementary figure 2: Significant (a) KEGG pathway terms, (b) GO Biological Process, and (c) GO Molecular Function associated with the overlap downregulated gene targets of mRNAs for the

main effects (CON vs RES) for fetal muscle. The size and color of the dots represent the number of genes and the range of statistical significance, respectively. The red color indicates higher -log10(FDR) values, followed by pink, purple and blue colors. The y-axis represents the GO and KEGG terms, and the x-axis represents the fold enrichment. The *P* values were corrected for multiple tests using the false discovery rate (FDR) ≤ 0.05. The enrichment tests were performed using ShinyGO tools (http://bioinformatics.sdstate.edu/go/)

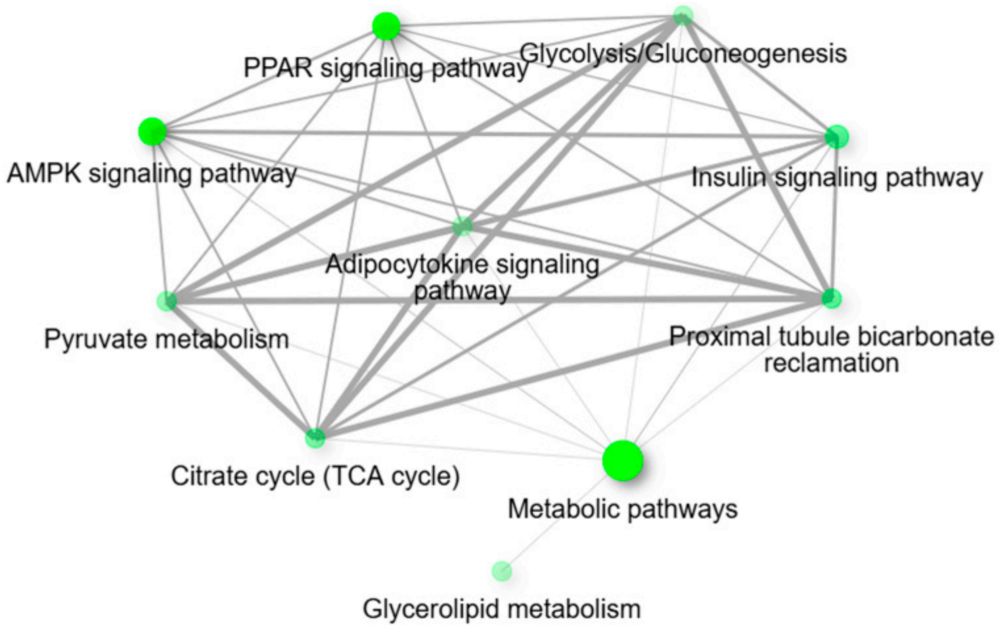

Supplementary figure 3: Interactive plot showing the relationship between enriched pathways (at an Enrichment FDR ≤ 0.05) for the main effects (NoOCM vs OCM) for fetal muscle. Two pathways (nodes) are connected if they share 20% or more genes. Darker nodes are more significantly enriched gene sets. Bigger nodes represent larger gene sets. Thicker edges represent more overlapped genes. The network constructions were performed using ShinyGO tools (http://bioinformatics.sdstate.edu/go/).

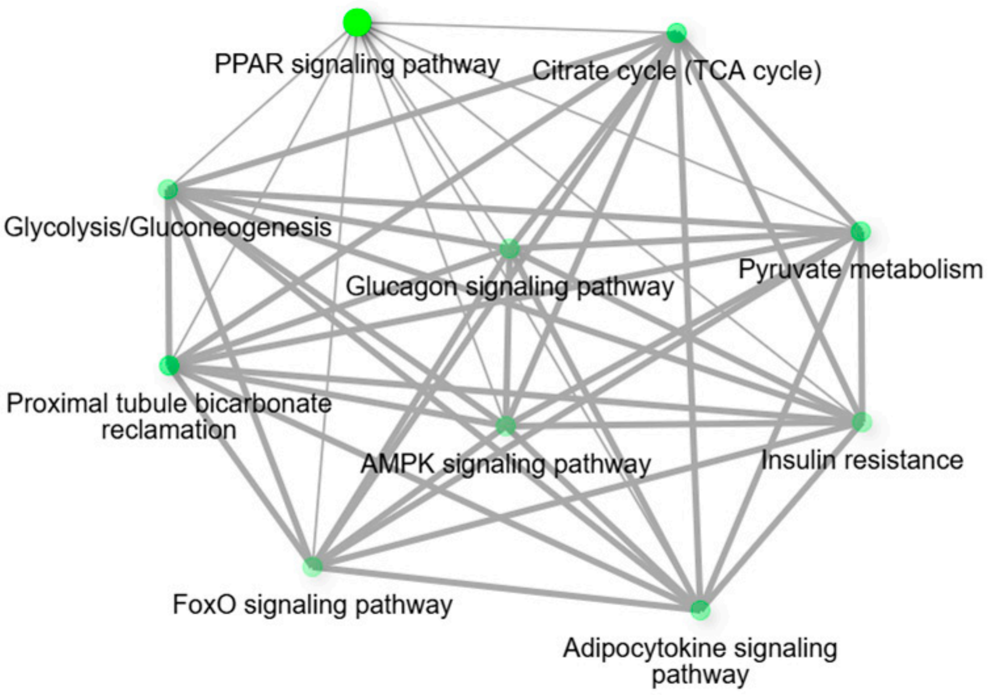

Supplementary figure 4: Interactive plot showing the relationship between enriched pathways (at an Enrichment FDR ≤ 0.05) for the main effects (CON vs RES) for fetal muscle. Two pathways (nodes) are connected if they share 20% or more genes. Darker nodes are more significantly enriched gene sets. Bigger nodes represent larger gene sets. Thicker edges represent more overlapped genes. The network constructions were performed using ShinyGO tools (http://bioinformatics.sdstate.edu/go/).
